# Fatigue, Emotional Distress, and Illness Uncertainty in Patients with Metastatic Cancer: Results from the Prospective NEOETIC_SEOM Study

**DOI:** 10.3390/curroncol29120763

**Published:** 2022-12-08

**Authors:** Adan Rodriguez-Gonzalez, Veronica Velasco-Durantez, Carla Martin-Abreu, Patricia Cruz-Castellanos, Raquel Hernandez, Mireia Gil-Raga, Esmeralda Garcia-Torralba, Teresa Garcia-Garcia, Paula Jimenez-Fonseca, Caterina Calderon

**Affiliations:** 1 Department of Medical Oncology, Hospital Universitario Central de Asturias, ISPA, 33011 Oviedo, Spain; 2 Department of Medical Oncology, Hospital Universitario de Canarias, 38320 Tenerife, Spain; 3 Department of Medical Oncology, Hospital Universitario La Paz, 28046 Madrid, Spain; 4 Department of Medical Oncology, Hospital General Universitario de Valencia, CIBERONC, 46014 Valencia, Spain; 5 Department of Medical Oncology, Hospital General Universitario Morales Messenger de Murcia, 30008 Murcia, Spain; 6 Department of Medical Oncology, Hospital Universitario Santa Lucia, 30202 Cartagena, Spain; 7 Department of Clinical Psychology and Psychobiology, Faculty of Psychology, University of Barcelona, 08007 Barcelona, Spain

**Keywords:** quality of life, fatigue, emotional distress, uncertainty, cancer, antineoplastic treatment

## Abstract

A cancer diagnosis can have a substantial impact on a patient’s mental health and quality of life. The aim of this study was to investigate the prevalence of fatigue, emotional distress, and uncertainty and examine the predictive value they have on the quality of life of advanced cancer patients. A prospective, multicenter study was conducted between February 2020 and May 2021 of individuals diagnosed with an advanced, unresectable neoplasm prior to initiating systemic antineoplastic treatment. Participants completed questionnaires to quantify fatigue, emotional distress, disease uncertainty, and quality of life. A linear regression analysis was performed to study the predictive QoL variables. The study population comprised 508 patients, 53.7% of whom were male and had a mean age of 54.9 years. The most common cancers were digestive (40.6%), bronchopulmonary (29.1%), and breast (8.5%); the most frequent histology was adenocarcinoma (63%); and most were stage IV (79.7%). More than half (55.7%) suffered fatigue, and 47.7% exhibited emotional distress; both were more prevalent among women. Fatigue, emotional distress, and disease uncertainty all correlate with diminished quality of life. Similarly, ECOG performance status and the demographic variables of age, sex, and comorbidities impacted quality of life. This patient sample displayed a high prevalence of fatigue and emotional distress, together with illness uncertainty, which are clearly linked to waning quality of life. To decrease the experience of fatigue and improve mental health treatment in cancer patients, interventions based on a biopsychosocial model must be intensified.

## 1. Introduction

Cancer is a public health problem in our society with an ever-growing incidence, with estimates of some 21.6 million new cases by 2030 [1]. A major consideration when addressing this disease is the considerable negative repercussions for patients’ quality of life (QoL), due to the disease process itself, its treatment, and its duration [2]. Though not generally quantified, economic, social, and family aspects also affect their QoL but are not generally measured Such is the case from the very onset of disease, lasting throughout treatment and the final stage of illness [3].

WHO defined quality of life as the individual’s perception of the place they occupy in the cultural setting and in the system of values where they live, as well as with respect to their goals, expectations, criteria, and concerns; all tempered by their physical health, psychological state, degree of independence, social relations, environmental factors, and their personal beliefs [4]. Similarly, QoL can be conceptualized as a subjective perception that encompasses all the patient’s facets and needs, constantly evaluating the differences between the individual’s actual situation and their ideal situation at any given time. Thus, it is a dynamic path that changes over time [5,6].

Specifically, individuals with advanced cancer experience a variety of persistent, unpleasant, and highly limiting physical and psychological symptoms that negatively impact their QoL. These patients commonly exhibit weight loss, fatigue, and pain [7,8]. Depending on the type of tumor, certain symptoms prevail over others; thus, in people with lung cancer, dyspnea, fatigue, and cough are the most frequent [9], whereas in gastrointestinal neoplastic disease, fatigue, pain, and weight loss are the most prevalent [10]. Likewise, breast or gynecological cancers are accompanied most often by psychological symptoms, such as stress, depression, and sexual alterations, in addition to a high prevalence of fatigue [11,12].

Cancer-related fatigue is one of the most prevalent symptoms patients suffer both during and after treatment [13,14], affecting 50–90% of all cases [14,15,16]. Fatigue appears regardless of age, sex, type of cancer, stage of disease, and treatment modality [15,17,18] and can become persistent, thereby limiting QoL and the activities of daily life for years [13,14,16]. Moreover, cancer-associated fatigue is unlike the exhaustion that most people experience as a result of their daily activities, inasmuch as it is not proportional to the level of effort and rest or sleep fail to remedy it [14,18]. Not only does it impair QoL, but it also restricts the person’s physical and social activities and their ability to return to work [14,16].

Until recently, fatigue in patients with cancer was neglected, and more attention was given to symptoms such as nausea and pain [19,20]. One of the reasons why fatigue has gone unnoticed is the variety of factors that can contribute to its development and conceal it, such as what is known as the general cancer syndrome, the direct consequence of the neoplasm or its treatment on the central nervous system or muscle energy metabolism, sleep or circadian rhythms, stress, despondency or depression, immune activation, anemia, cachexia, or malnutrition, etc. [21,22,23]. Likewise, despite its high prevalence and possible interference with activities and patients’ wellbeing, research into the etiopathogenesis of and approaches to fatigue in patients with metastatic cancer is meager.

Furthermore, individuals with advanced cancer display a high prevalence of emotional distress that varies depending on age and type of cancer; nonetheless, overall, emotional problems are found in more than 30% of all oncological patients [24]. Depression and anxiety are common symptoms among patients with cancer, with rates ranging between 11% and 57% for depression and between 6.5% and 23% for anxiety [14,25,26]. These psychological issues have been found to be affected by 5 main symptoms: anxiety, depression, fear of dying, demoralization, and the inability to cope with the disease. All appear in relation to the strain of the treatment they must deal with and translate into worse QoL, worse functional status, increased suicide rates, and early death [27].

Uncertainty is another pressing aspect of cancer patients’ journeys and can cause them to experience a prolonged feeling of loss of control, having a direct, negative impact on how they cope with cancer, their psychological wellbeing, and QoL. In fact, cancer and the uncertainty surrounding its evolution are associated with psychological distress in up to 30–50% of the cases [15,25,28,29,30,31]. Some studies have revealed a prevalence of uncertainty surrounding the disease in up to 60% of the oncological population [32]. In patients with an advanced, unresectable cancer, uncertainty is the wellspring of the inability to predict the course of the neoplasm with any certainty, of the perception of the future as threatening, of the advanced status of the process, and/or of communication barriers (conspiracy of silence, emotional blockage, cultural level, the healthcare professional’s ability, etc.). Numerous studies have linked clinical uncertainty with diminished QoL and resilience, anxiety, depression, and other negative effects.

Given the widespread presence of cancer, those factors that, from the very beginning of the disease, might be associated with impaired QoL must be identified. This will make it possible to plan actions that maximize the factors that can positively affect patients’ QoL so as to prevent, eliminate, or minimize those that contribute to its worsening.

In this context, the objective of this study is to analyze the prevalence of fatigue, emotional distress, and uncertainty in patients with metastatic cancer and to examine the predictive value of fatigue, emotional distress, and uncertainty in these patients’ QoL. This analysis has been conducted in a sample of individuals with recently diagnosed, advanced cancer prior to initiating antineoplastic treatment to determine the prevalence of these symptoms at the beginning of the disease, as well as how they relate to the decline in QoL these individuals display at the beginning of their cancer journey. We expect to find high levels of fatigue, emotional distress, and uncertainty among the participants, and that the factors explored in this study account for a portion of the variance in QoL.

## 2. Materials and Methods

### 2.1. Study Design and Population

This study is part of the prospective, consecutive NEOetic registry executed in 15 hospitals in Spain between February 2020 and May 2021. After attaining informed consent in writing, all the participants that were treated at the medical oncology departments at these centers and had a confirmed diagnosis of an advanced neoplasm were invited to participate at their first appointment with the oncologist, during which they were informed of their diagnosis and the antineoplastic treatment to be administered.

Patients eligible for surgery or other therapies with curative intent, those whose physical condition, comorbidities, and/or age comprised a contraindication for antineoplastic treatment in the opinion of the treating oncologist, anyone who had been treated for another advanced cancer in the previous two years, or whose underlying personal, family, sociological, and/or medical condition might hinder their ability to participate in the study, were excluded. This investigation was conducted in accordance with the standing ethical principles and received prior approval by the ethical review boards of each institution and by the Spanish Agency for Medicines and Medical Devices (AEMPS; ID Code: ES14042015). The study involved filling out several questionnaires and gathering clinical data from the individual’s interview and their medical record. The procedures for collecting data were similar at all the hospitals, and the data regarding the participants were obtained from the centers where they received treatment. Participation was voluntary, anonymous, and in no way affected patient care. Data were collected and updated by the medical oncologist by means of a web platform (www.neoetic.es, accessed on 12 June 2021).

### 2.2. Description of Variables

Sociodemographic characteristics were collected using a standardized self-report form. Information concerning the subjects’ disease was obtained by the attending oncologist by reviewing their medical record. The person’s general status was assessed as per the Eastern Cooperative Oncology Group (ECOG) performance scale, with values ranging from zero (asymptomatic) to five (deceased). Any value was admitted as long as the oncologist deemed the patient eligible to receive systemic treatment. The oncologist gave the participants the questionnaire during the course of the appointment, and the patient filled it out at home prior to beginning systemic cancer treatment. 

Quality of life was measured with the QLQ-C15 PAL questionnaire [33], which comprises 15 items for the assessment of two multi-item functional scales, two symptom scales with multiple items each, five single-item symptom scales, and a question on global health status. For the purposes of this study, items 7 and 11 were eliminated, as they are similar to the one that refers to fatigue. This scale has been validated in multiple languages, including Spanish [34]. The total scale score ranges from 0 to 100; the higher the score, the better their QoL. In this sample, Cronbach’s alpha for the scale was 0.87.

Fatigue was gauged using the three-item Health-related QoL (HRQoL) fatigue scale and the EORTC QoL QLQ-C30 questionnaire (version 3.0), which has been validated in Spanish [35,36]. The questionnaire consists of three simple questions: “Did you need rest?”, “Have you felt weak?”, and “Were you tired?”. The items were converted to a rating scale from 0 to 100. According to the thresholds of clinical importance (TCIs), the values recommended by Giesinger et al. [37] for the fatigue scale were ≥39. In this study, Cronbach’s alpha for the scale was 0.88.

Emotional distress over the past 7 days was determined by the 18 items of the Brief Symptom Inventory (BSI), one of the most commonly used instruments for this purpose [38]. Raw scores are converted to T-scores based on gender-specific, normative data. To identify individuals with significant levels of emotional distress, the BSI applies the clinical case rule [38], originally developed for SCL-90. Based on the cut-off values recommended by Derogatis [38], patients whose T score ≥67 were deemed to suffer “possible emotional distress”. The Spanish version of the BSI has demonstrated good reliability and validity among Spanish cancer patients [39]. Cronbach’s alpha varied from 0.81 to 0.90 [38].

Uncertainty of Illness was computed by means of the 5-item Mishel Uncertainty of Illness Scale (MUIS) validated for the Spanish population [40,41,42]. This questionnaire appraises reactions to uncertainty, ambiguity, and the future. Items are scored on a Likert scale ranging from 1 (the patient does not exhibit any of the characteristics described in the item at all) to 5 (the patient displays the highest degree of the described characteristic), yielding possible scores of 5 to 25, with higher scores corresponding to greater uncertainty. Patients whose T score ≥16 were deemed to suffer “uncertainty”. Cronbach’s alpha was 0.83 [40].

The questionnaires were given to the participants after the study was explained to them and they agreed to participate and signed the informed consent form, and after shared oncologist-patient decision making regarding systemic oncological treatment for incurable, advanced cancer. Patients completed the questionnaires at home and gave them to their oncologist at their following appointment, coinciding with the start of antineoplastic treatment.

### 2.3. Statistical Methods

Descriptive statistics, means, and standard deviations (SD) were calculated for the sample’s demographic and clinical characteristics. A bivariate chi-square was used to examine differences between fatigue and emotional distress according to sex and disease stage. Pearson’s correlation coefficient was calculated to gauge the association of QoL with fatigue, emotional distress, and disease uncertainty. Multicollinearity across variables was rejected by the variance inflation factor being <5 for all and the tolerance >0.2 [43]. To ascertain the predictive variable of QoL, a two-block linear regression model was carried out. In the first block, fatigue, emotional distress, and disease uncertainty were recorded as criterion variables. In the second block, sex and age were entered as independent variables. We applied R-squared and Cohen’s standardized f2 measure of effect size to interpret the data [44]. For all analyses, significance was set at α < 0.05. Statistical analyses were performed with Statistical Package for Social Sciences (SPSS) software, version 25.0 (IBM SPSS Statistics for Windows, Armonk, NY, USA: IBM Corp.).

## 3. Results

### 3.1. Population Description

This study population consisted of 508 patients, of whom 273 (53.7%) were male, the mean age was 54.9 years (SD = 10.1), and 49.6% were elderly (>70 years). Most were married or had a partner (83.1%) and/or children (83.7%). Less than half (47.8%) had a primary education level. As for employment status, all were retired or unemployed. The most common cancers were digestive (40.6%), bronchopulmonary (29.1%), and breast (8.5%). The most common histology was adenocarcinoma (63%) and most neoplasms were stage IV (79.7%); the remaining were unresectable stage III. The most frequently administered treatments were chemotherapy (55.7%), chemotherapy with targeted therapy (10.6%), and chemotherapy with immunotherapy (9.6%). In 26.2% of participants, the treatment decision was informed by the presence of a molecular biomarker. Estimated survival was <18 months in 48.8% of the sample (see Table 1).

### 3.2. Prevalence of Fatigue and Emotional Distress

All told, 283 (55.7%) subjects expressed experiencing fatigue (score ≥ 39), as per Giesinger et al. [37]. Females exhibited more fatigue than males (χ^2^ = 11.689; *p* < 0.001). Emotional distress was present in 47.7% of the population (T score ≥ 67), according to the cut-off values recommended by Derogatis [38]. Women displayed greater emotional distress than men (χ^2^ = 6.347; *p* = 0.012) (see Table 2). Uncertainty was present in 36.4% of the study population (PD ≥ 16). Patients with other comorbidities (such as chronic illness or psychiatric disorder) displayed greater uncertainty than those with comorbid cardiovascular disease (χ^2^ = 7.150; *p* = 0.007); patients with a worse ECOG presented more uncertainty (χ^2^ = 9.155; *p* = 0.027). No differences were detected with respect to the incidence of fatigue, emotional distress, or uncertainty across stages or comorbidities (see Table 2).

### 3.3. Relationship of Fatigue, Emotional Distress, and Uncertainty of Illness with Quality of Life

In an initial analysis of bivariate correlations, fatigue, emotional distress, disease uncertainty, and ECOG performance status were seen to be significantly associated with QoL (correlations of −0.23 to −0.71, all *p* < 0.001; Table 3). A linear regression analysis contemplating fatigue, emotional distress, illness uncertainty, and ECOG status as predictors, together with the demographic variables of age, sex, and comorbidities, evinced a significant (statistical, non-causal) influence of fatigue, emotional distress, illness uncertainty, and ECOG status on QoL (F = 129.50, *p* <0.001). This model revealed high explanatory power (adjusted *R*^2^ = 0.61 for the model), in keeping with Cohen’s guidelines [44] (f2 = 1.60). More symptoms on these scales were associated with worse QoL (see Table 4).

## 4. Discussion

In this study, we have confirmed a high prevalence of fatigue (55.7%) and emotional distress (47.7%) among individuals with advanced, unresectable cancer prior to the start of cancer treatment. Fatigue, emotional distress, and uncertainty about illness were associated with impaired QoL. The clinical variable, ECOG performance status, and the demographic variables of age, sex, and comorbidities also negatively influenced QoL.

Fatigue is widespread among oncological patients, despite the disparity in prevalence rates reported (ranging from 30–99%) [45,46,47,48,49,50]. In one study carried out in Italy with a sample of 1394 patients with cancer, fatigue was reported in 62% of the cases, severely impacting QoL and, for one of every three patients, hindering their daily activity [51]. This study has examined information from a total of 508 patients from different centers in Spain and determined a 54% fatigue prevalence rate. The Italian series reveals a higher prevalence rate, although it is likely due to how participants were included in the sample and the fact that they were evaluated without regard for disease timing or stage. In this Spanish series, we have examined fatigue among the patients who are going to receive systemic treatment upon diagnosis of an advanced, unresectable disease. In light of the disparate patterns of how different types of cancer evolve, the more protracted survival and follow-up are, the greater the likelihood of fatigue.

We detected no statistically significant differences between patients with fatigue and the same ECOG status based on comorbidities or between locally advanced and metastatic disease in this series. Bearing in mind that the study was conducted prior to initiating antineoplastic treatment, this is probably due to cancer-related immune/inflammatory, metabolic, neuroendocrine, and genetic biomarkers [48] that have an equal effect regardless of the individual’s overall status. 

Likewise, our series revealed a prevalence of 48% psychological distress, which is more common among women than men (52% vs. 47%) and higher than the 30–50% reported in the literature [25,28,29]. This divergence may be attributable to the sample characteristics (proportion in terms of sex, age, or tumor stage) [52], to the diversity of instruments used in the studies, and to the timepoint at which our series was evaluated, immediately after diagnosis and just before starting systemic treatment. As with fatigue, these data are not affected by the number or type of comorbidities. Several different studies have shown that being female, together with advanced disease, is one of the greatest risk factors for developing psychological distress, be it in the form of depression or anguish/anxiety [53,54,55,56]. In terms of age, despite the fact that elderly people predominate in our sample and country (Spain), we have found more psychological distress in younger participants, as also reported in other series [45].

Our study has shown that fatigue, psychological distress, uncertainty, and ECOG performance status are associated with diminished QoL in people with metastatic cancer prior to initiating systemic treatment. To the best of our knowledge, this is the first study to examine these factors as a whole and their correlation with QoL. Our results indicate that 61% of the variance in QoL can be attributed to these factors. The greater the fatigue, psychological distress, uncertainty, and worse ECOG, the worse the patient’s QoL will be. Of all these factors, fatigue accounts for 49% of the decline in QoL, and psychological distress accounts for another 10%.

These results entail compelling clinical implications. As this study evidences, fatigue is one of the most common symptoms among individuals with cancer and can persist for months or even years after treatment [57]. Based on other recent studies, incorporating specific guidelines for physical activity before, during, and after treatment for these patients could decrease fatigue, which, as we have seen here, impairs QoL [58,59,60]. Through this work, the authors have detected a high prevalence rate of emotional fatigue that several researchers have associated with a reduction in the efficacy of and tolerance to treatment in oncological patients [61]. Consequently, it would be wise to integrate a psychological evaluation into medical oncology consultations and, if necessary, refer the individual to a mental health specialist.

This study has several limitations. The first is that it included patients with different tumor types that we have not been able to compare to each other; consequently, we cannot extrapolate these data to particular types of metastatic neoplasms. The second is that the study was conducted among the Spanish cancer population, and care must be exercised when transferring our results to other countries, especially non-Western countries, inasmuch as cancer care depends on the organization of the country’s healthcare system and economy and Spain offers universal access to public healthcare. The third limitation is that, given the cross-sectional design of this study, with patients’ being evaluated after diagnosis and before beginning treatment, causality of the associations cannot be inferred, nor can the variability of the prevalence of fatigue and emotional distress over the course of advanced disease be ascertained. Future studies must assess the causal relationship between fatigue, psychological distress, and uncertainty and QoL in individuals with cancer at several timepoints to determine the changes in QoL and what may be causing them.

## 5. Conclusions

In short, this prospective, multicenter study reveals that there is a high prevalence of fatigue and psychological distress upon diagnosis of advanced, unresectable cancer and that they impact patients’ QoL. Similarly, a high prevalence of uncertainty surrounding the disease has been found; nevertheless, we have been unable to demonstrate that this is independently related to decreased QoL. This should prompt oncologists to be more interested in studying and treating the fatigue, emotional distress, and uncertainty surrounding their disease that tend to go unnoticed or are underappreciated. 

## Figures and Tables

**Table 1:** Cancer types, stages, and medical comorbidities of patients (*n* = 508).

Variables	N	%
**Sex**		
Female	235	46.3
Male	273	53.7
**Age (years)**		
<45	17	3.4
45–70	239	47.0
>70	252	49.6
**ECOG**		
0	174	34.3
1	300	59.1
2	31	6.1
3	3	0.6
**Comorbidities**		
Cardiovascular disease	206	40.6
Chronic illness	11	2.2
Psychiatric disorder	21	4.1
Cardiovascular + chronic disease	34	6.7
Cardiovascular + chronic disease + psychiatric disorder	17	3.3
Obesity (body mass index ≥30)	80	15.7
Weight loss	69	13.6
Other comorbidities	70	13.8
**Marital status**		
Married or partnered	335	83.1
Single/divorced/widowed	68	16.9
**Educational level**		
Primary	243	47.8
High school or more	265	52.2
**Children**		
With children	425	83.7
Without children	83	16.3
**Tumor site**		
Broncho-pulmonary	148	29.1
Digestive	206	40.6
Gynecological and breast	43	8.5
Others	111	21.9
**Histology**		
Adenocarcinoma	320	63.0
Others	188	37.0
**Stage**		
Locally Advanced	103	20.3
Metastatic Disease (IV)	405	79.7
**Biomarker for treatment decisions**		
No	375	73.8
Yes	133	26.2
**Survival**		
<18 months	248	48.8
≥18 months	260	51.2
**Type of treatment**		
Chemotherapy	283	55.7
Chemotherapy and immunotherapy	49	9.6
Chemotherapy and targeted drug	54	10.6
Immunotherapy	35	6.9
Targeted drug	28	5.5
Others	58	11.4

**Table 2:** Fatigue and emotional distress across sexes and stage types.

Variables	Fatigue		Emotional Distress		Uncertainty	
	None (FA < 38.9)	Fatigue (FA ≥ 39)	None (ED < 66.9)	Em. Distress (ED ≥ 67)	None (UN < 15.9)	Fatigue (UN ≥ 16)
Sex (*n*, %)						
Male	140 (62.2)	133 (47.0)	155 (58.7)	113 (47.5)	172 (53.3)	101 (37.0)
Female	85 (37.8)	150 (53.0)	109 (41.3)	125 (52.5)	151 (46.7)	84 (45.4)
*p*-value	<0.001 *		0.012 *		0.770	
Stage type (*n*, %)						
Locally advanced	40 (17.8)	63 (22.3)	57 (21.6)	45 (18.9)	61 (18.9)	42 (22.7)
Metastatic	185 (82.2)	220 (77.7)	207 (78.4)	193 (81.1)	262 (81.1)	143 (77.3)
*p*-value	0.212		0.577		0.303	
Comorbidities (*n*, %)						
Cardiovascular disease	112 (49.8)	142 (50.2)	131 (49.6)	119 (50.0)	176 (54.5)	78 (42.2)
Others	113 (50.2)	141 (49.8)	133 (50.4)	116 (50.0)	147 (45.5)	107 (57.8)
*p*-value	0.929		0.932		0.007 *	
ECOG (*n*, %)						
0	104 (46.2)	70 (24.7)	101 (38.3)	70 (29.4)	121 (37.5)	53 (28.6)
1	113 (50.2)	187 (66.1)	151 (57.2)	147 (61.8)	187 (57.9)	113 (61.1)
2	7 (3.1)	24 (8.5)	10 (3.8)	20 (8.4)	13 (4.0)	18 (9.7)
3	1 (0.4)	2 (0.7)	2 (0.8)	1 (0.4)	2 (0.6)	1 (0.5)
*p*-value	0.001 *		0.046 *		0.027 *	

FA = Fatigue; ED = Emotional Distress. * These values indicate significance at the 5% level.

**Table 3:** Correlations of fatigue, emotional distress, and illness uncertainty with QoL.

Variables	Fatigue	Emotional Distress	Illness Uncertainty	ECOG	Quality of Life
Fatigue	1				
Emotional distress	0.559 **	1			
Illness uncertainty	0.177 **	0.264 **	1		
ECOG	0.237 **	0.129 **	0.105 *		
Quality of life	−0.705 **	−0.645 **	−0.298 **	−0.251 **	1

** *p* < 0.001; * *p* < 0.005.

**Table 4:** Linear regression models probing statistical predictor of QoL.

	Quality of Life				
Predictor	Estimate	*R* ^2^	t	*p*-Value	Cl
(Intercept)	116.682		17.852	0.001 *	103.8–129.5
Fatigue	−0.275	0.49	−13.551	0.001 *	−0.31–−0.23
Emotional distress	−0.869	0.59	−9.862	0.001 *	−1.0–−0.69
Illness uncertainty	−0.546	0.60	−3.896	0.001 *	−0.82–−0.27
ECOG	−2.556	0.61	−2.711	0.001 *	−4.4–−0.70
Sex: male	−0.390		−0.360	0.719	−0.13–0.07
Age	−0.027		−0.493	0.622	−2.50–1.40
*R*^2^ adjusted total		0.61			

* These values indicate significance at the 5% level.

## Data Availability

The datasets generated during and analyzed during the current study are not publicly available for reasons of privacy. They are however available (fully anonymized) from the corresponding author on reasonable request.
